# Zn/La Mixed Oxides Prepared by Coprecipitation: Synthesis, Characterization and Photocatalytic Studies

**DOI:** 10.3390/ma13214916

**Published:** 2020-10-31

**Authors:** Amalia Maria Sescu, Maria Harja, Lidia Favier, Laurence Oughebbi Berthou, Consuelo Gomez de Castro, Aurel Pui, Doina Lutic

**Affiliations:** 1 Department of Chemical Engineering, Faculty of Chemical Engineering and Environmental Protection, “Gheorghe Asachi” Technical University of Iasi, 73, Prof.dr.doc. D. Mangeron Blvd., 700050 Iasi, Romania; sescu.amaliamaria@gmail.com; 2 Ecole Nationale Supérieure de Chimie de Rennes, Univ Rennes, CNRS, ISCR—UMR6226, F-35000 Rennes, France; laurence.oughebbi@ensc-rennes.fr; 3 Department of Materials and Chemical Engineering, Faculty of Chemical, Complutense University of Madrid, Av. Séneca, 2, 28040 Madrid, Spain; cgcastro@quim.ucm.es; 4 Department of Chemistry, Faculty of Chemistry, “Alexandru Ioan Cuza” University of Iasi, Bvld. Carol I No 11, 700506 Iasi, Romania; aurel@uaic.ro

**Keywords:** lanthanum, mixed oxides, photocatalytic performance, photocatalyst reuse, zinc

## Abstract

Mixed oxides containing zinc and lanthanum were prepared by coprecipitation in alkaline medium, followed by calcination at 400 °C. The initial precipitation product and the calcined form were characterized by Brunauer–Emmett–Teller (BET) method adsorption of nitrogen at −196 °C, Scanning Electron Microscopy/Electron-Probe Microanalysis (SEM/EPM), Ultraviolet—Diffuse Reflectance Spectroscopy (UV-DRS) and Infrared (IR) spectroscopy. The band gap slightly changes from 3.23 eV to 3 eV by calcination. The photocatalytic performance of the solids were investigated in diluted aqueous medium, by using clofibric acid (CA), a stable and toxic molecule used as precursor in some pesticides and drugs, as test compound, possibly found in the wastewaters in low concentrations. The effects of the degradation extent, determined by high performance liquid chromatography (HPLC) and total organic carbon (TOC) measurements, were investigated at different initial concentrations of CA. Within about 60 min the CA degradation is almost total at low concentration values (3 ppm) and reaches over 80% in 180 min for an initial concentration of 50 ppm. Moreover, the CA removal performance of photocatalyst remains excellent after three cycles of use: the removal yield was practically total after 60 min in the first two cycles and reached 95% even in the third cycle.

## 1. Introduction

The scientists’ concern for the green elimination of various persistent organic contaminants from wastewaters resulted, among others, in a high interest paid for obtaining highly performant materials for the photocatalytic reactions mediated by semiconductive solids, by oxidation reactions [1,2,3,4]. This class of reactions belong to the so-called AOPs (Advanced Oxidation Process), being very popular especially for the purification of wastewaters from low amounts of pesticides, drug compounds, dyes, disinfectants and other very stable and non-biodegradable chemicals, which depreciate the water quality, despite their ppm-range concentrations [5,6]. A photocatalytic process supposes the capturing of light energy (UV or visible range), mediated by a semiconductive material, generating an electron-hole pair, by the promotion of an electron from the valence band to the conduction band. This step in turn initiates a series of reactions with the participation of water and dissolved oxygen and generates HO∙ radicals and other active oxidizers [7,8]. The adsorption of a POP (Persistent Organic Pollutant) on the surface of a photocatalyst renders possible the joint between this species and oxidant species, resulting in the gradual decomposition of the compound, up to the total mineralization to CO_2_ and H_2_O, in an ideal situation [1,2,9,10].

The most used material in photocatalysis nowadays is titanium dioxide. However, a high number of studies are dedicated for obtaining also other materials with higher activity, stability, possibility of reuse or other advantages with regard to TiO_2_ [2,11]. In this respect, zinc oxide, tin oxide, gallium oxide, iron oxide, wolfram oxide, cadmium sulfide, sodium tantalate and combinations thereof are just some often presented examples to illustrate the availability of a high number of materials with interesting properties in the photocatalytic applications [12,13]. As an example, zinc oxide, is recognized as a better n-type semiconductor because of its wide band gap energy of 3.37 eV as well as its large surface-to-volume ratio, showing a high excitation binding energy (60 meV) at ambient temperature, compared to TiO_2_ [12]. In recent years, the doping of ZnO with noble metals is often mentioned as a smart efficient way to improve either the electro-hole pair formation or their recombination rate reduction enhancing light absorption and thus its photocatalytic reactivity [6,9].

There are seldom studies concerning the use of lanthanum in the formulations of semiconductive oxides used as photocatalysts [14,15,16].

Despite its high band gap value in pure bulk state (about 5.5 eV), the use of lanthanum for doping other semiconductive oxides led to the improvement of the photocatalytic behavior due to the band gap decrease in the nanoparticle size range [17,18,19]. The above mentioned papers [17,18] deal with zinc oxide doping with lanthanum oxide and claim that this doping had a strong effect of improvement of the (photo)catalytic properties, despite the fact that lanthanum (atomic number 57) is essentially bulkier than Zn, in both ionic and covalent compounds [20] (Table 1).

In this work, we report an extremely simple and cheap preparation method for obtaining of Zn-La mixed oxide, by the coprecipitation of a mixture of soluble salts of zinc and lanthanum, by using NaOH solution, at a constant pH value of 10. Part of the resulted product (labeled Zn4La) was subjected to slow heat treatment at 400 °C, to obtain the corresponding mixed oxide (labeled Zn4La400). Both solids were fully characterized by different methods such as diffuse reflectance spectroscopy (DR) and infrared spectroscopy (IR) for the surface analysis, pure nitrogen adsorption at −196 °C (BET) for the investigation of the porous structure and by scanning electron microscopy (SEM) to highlight the solid morphology. The Energy Dispersive X-ray Analysis (EDAX) was used to show the homogeneity of the chemical composition of the samples. Subsequently, the two solids were used for the first time as photocatalytic materials for the degradation of clofibric acid (CA), a toxic compound which can reach the wastewaters either from applications in agriculture for controlling the plants-growing process (produces grow inhibition) or from the transformation of the drug clofibrate (a cholesterol lowering agent) in the human body.

Both solids have quite high photocatalytic activity. The calcined product is superior to the as-synthesized precipitated sample, therefore, a detailed study of its photocatalytic activity was carried out. A special attention was paid to the investigation of the effects of some relevant process parameters such as the initial pollutant content and catalyst load, as well as to the evaluation of its photocatalytic performance by reusing the same solid in several reaction cycles.

## 2. Materials and Methods

### 2.1. Materials Synthesis and Characterization

**Figure 1:**
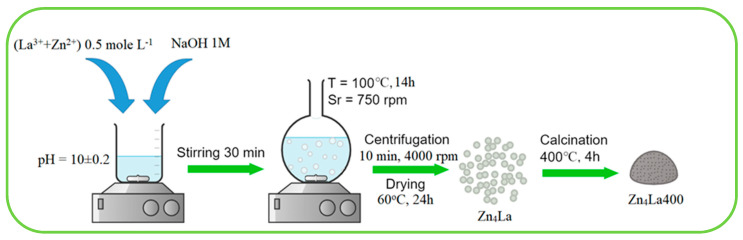
Schematic presentation of the main preparation steps used for synthesis of Zn_4_La mixed oxides.

The used chemicals were of analytical grade and were employed as received. Zinc nitrate, lanthanum acetate and sodium hydroxide from Sigma-Aldrich (St. Louis, MO, USA) were used. The preparations were made using distilled water and 500 mL of precursor salts solution were prepared by mixing individual solutions of the two salts such to contain an overall metals concentration of 0.5 mol/L and a Zn/La molar ratio of 4/1 (Figure 1). This solution mix was slowly added in a beaker under continuous magnetic stirring, by peristaltic pump, setting the flow rate at 10 mL/min in the same time to the proper flow addition of NaOH solution (1 M), regulated to keep the pH value of the medium at 10 ± 0.2. In order to settle the due pH value from the very beginning of the reagents addition, an initial volume of 30 mL of water was placed in the beaker before dropping the metallic salts mixture and NaOH solution. After ending the reactants addition, the mixture was aged at ambient temperature for 30 min under continuous stirring, then placed under reflux on an oil heating bath at 100 °C under intense stirring (750 rot/min), for 14 h. After cooling, the product was retrieved by centrifugation (10 min at 4000 rpm), washed five times using distilled water and then dried for 24 h at 60 °C. Half of the obtained solid was calcined at 400 °C (heating rate 1°/min) for 4 h (Figure 1).

The X-ray diffraction (XRD) spectra were obtained on a Shimadzu D6000 device (Shimadzu Corporation, Tokyo, Japan), in the range 20–80°, using CuKα radiation (λ = 1.5406 Å). The IR spectra were traced on Jasco FT/IR-6100 spectrometer (Jasco, Waltham MA, USA). The porosity analysis was investigated by BET adsorption of pure nitrogen at −196 °C on a Quantachrome machine Nova 2200e (Quantachrome Instruments, Graz, Austria); the dedicated software was employed to determine the BET surface area, free pore volume determination and BJH pore size distribution. The sample morphology characterization was done on a scanning electron microscope (JEOL JSM-7100, Jeol Ltd., Tokyo, Japan) equipped with an EDS detector. For these measurements, an acceleration voltage of 10 kV was used.

### 2.2. Photocatalytic Tests

The photocatalytic assays were run in a cylindrical glass reactor of 1 L equipped with a central quartz tube a mercury vapor lamp (Osram UV-A, (OSRAM GmbH, Garching, Germany) 9W, presenting a maximum emission at 365 nm), under magnetic stirring (450 rpm). The reactions occurred at ambient temperature, at the native pH of the CA solution and at the maximal irradiation conditions (9.52 mW/cm^2^). Each day the CA solutions were prepared by simple dissolution of proper amounts of CA in ultrapure water. Before the irradiation, the adsorption-desorption equilibrium was let to settle, by agitating the aqueous suspension of photocatalyst and CA in dark for 30 min. For the photocatalytic process, irradiation time durations of up to 180 min were employed. Suspension samples were withdrawn at certain reaction time values, separated by filtration through filters with 0.45 µm porosity attached to a syringe, for complete separating of the solid before measuring the CA content. 

The CA concentrations from the solutions were measured by HPLC (Milford, MA, USA) on a WATERS^®^ system endowed with a photodiode array detector (WATERS™ 996, Milford, MA, USA). The mobile phase was a mixture of 60% acetonitrile and 40% ultrapure water. The detection consisted on the measurement of the absorbance at 227 nm; the retention time for CA was around 6.4 min. The CA mineralization level was expressed as the total organic carbon (TOC), performed on a Shimadzu TOC-5000-A instrument (Shimadzu Corporation, Kyoto, Japan). The average value of three measurements was recorded in the presented data.

The degree of CA degradation was calculated according to the relation:

(1) $$Degradation,\;\%=\frac{C_{0}-C_{t}}{C_{0}}\cdot 100,$$

where *C*_0_ and *C_t_* are, respectively, the CA concentrations at time zero and at time *t*.

For the determination of the extent of mineralization of clofibric acid during the photocatalytic process the following equation was used:

(2) $$Mineralization,\;\%=\frac{\left[TOC_{0}\right]-\left[TOC_{t}\right]}{\left[TOC_{0}\right]}\cdot 100,$$

where $\left[TOC_{0}\right]$ is the initial concentration of *TOC* (mg C/L) of the aqueous solution and $\left[TOC_{t}\right]$ is the *TOC* in mg C/L at measured after a reaction time t. 

## 3. Results and Discussion

### 3.1. Structural and Morphologic Characterization

#### 3.1.1. Powder XRD

**Figure 2:**
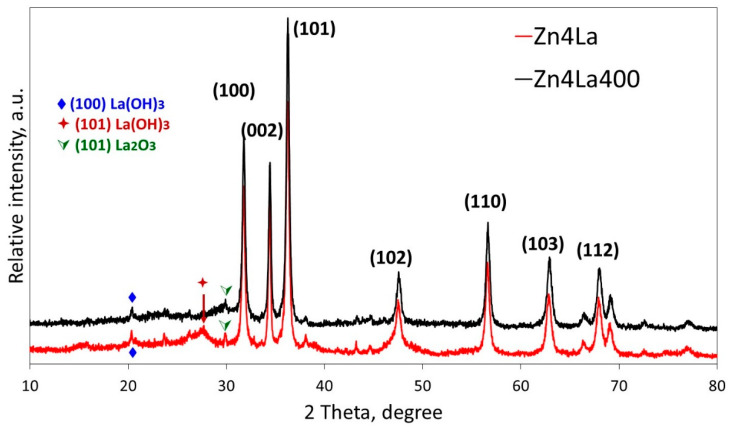
X-ray diffraction (XRD) patterns of samples Zn4La and Zn4La400.

The XRD patterns reveal that both as-synthesized and calcined samples display mostly the characteristic peaks due to zinc oxide [21,22,23]. Indeed, intense and narrow maxima appear at 31.8, 34.4, 36.3, 47.9, 56.6, 63 and 68°, assigned to the (100), (002), (101), (102), (110), (103) and (112), respectively, as filed in (Joint Committee on Powder Diffraction Standards—JCPDS: 89-7102). Some additional small peaks served for the identification of La(OH)_3_ phase by the presence of peaks at 20 and 28°, due to (100) and (101) planes and of the La_2_O_3_ highlighted by the peak from 30.4°, assigned to the (101) plane (Figure 2).

The same allure of the XRD spectra was obtained by Maninkidan et al. [17] on ZnO doped with up to 7% La, though the La to Zn ratio in the preparation was higher in our case. Therefore, investigations were necessary to investigate the samples composition and find out the reason for the presence of less lanthanum solids detectable by XRD in our case. It should be noticed that, the ZnO XRD pattern as well as SEM (JSM-7100, Jeol Ltd., Tokyo, Japan) images of pure ZnO prepared following a similar protocol as the one used in this work are available in the previous study of Lutic et al. [22] and the obtained results clearly showed that the morphology of pure ZnO was really very different than that of the material obtained by coprecipitation of Zn and La.

#### 3.1.2. SEM/EDS Analysis

**Figure 3:**
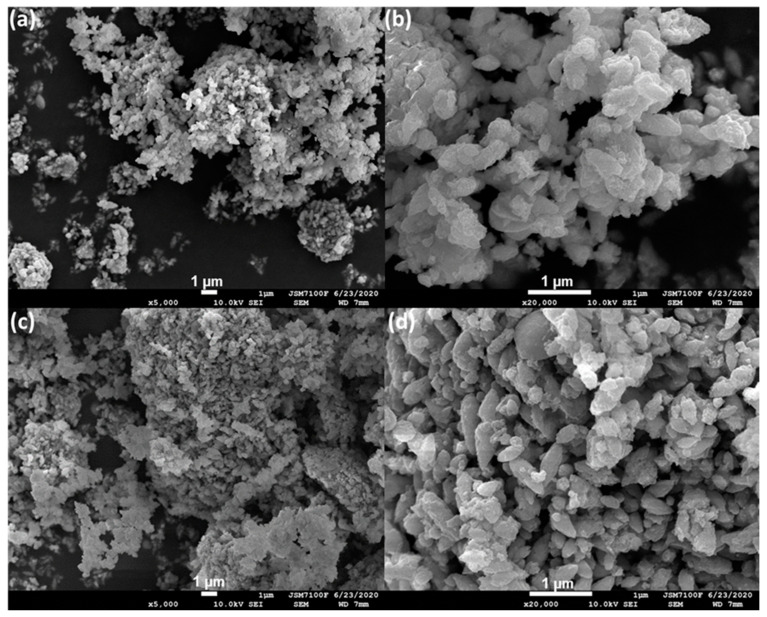
Representative scanning electron microscopic (SEM) images of the samples, (**a**) Zn4La—5000×, (**b**) Zn4La—20,000×, (**c**) Zn4La400—5000× and (**d**) Zn4La400—20,000×.

The SEM images of Zn4La and Zn4La400 are shown in Figure 3.

The morphology of the samples consists of platelets or flakes more or less compact, sometimes stacked as packing of lamellas. This morphology is quite similar to that of layered double hydroxides containing divalent and trivalent ions of hydrotalcite type [24,25]. For the Zn4La sample, the particles of quite uniform shapes and sizes are seen to be formed by stacking of numerous platelets with irregular perimeters, generating formations looking as rice grains of about 0.3–0.5 micrometers. After calcination, the Zn4La400 sample displays more particles quite heterogeneous in size and rough external surface, some of the keeping the rice-like shape and quite many others looking as broken stones.

**Figure 4:**
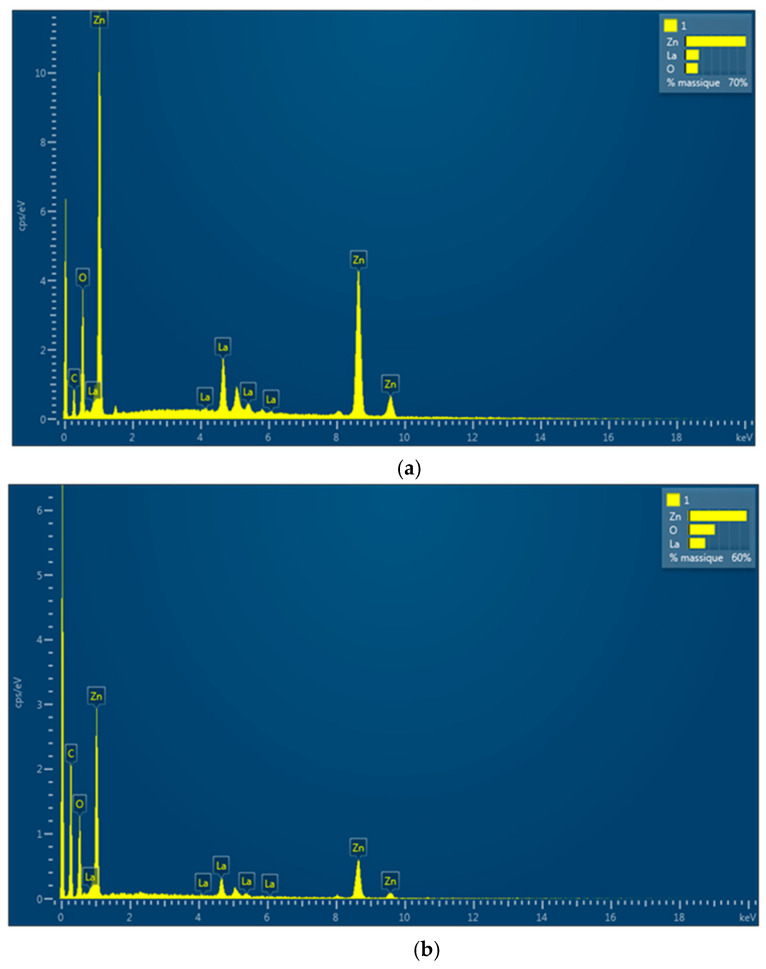
Energy Dispersive X-ray spectroscopy (EDS) analysis of the samples. (**a**) Zn4La (Zn:La = 1:0.1384); (**b**) - Zn4La400 (Zn:La = 1:0.1057).

The Energy Dispersive X-ray spectroscopy (EDS) analysis of the solids was performed to investigate the incorporation extent of lanthanum in the precipitation product and the method delivers information about the situation from the external layer of the solids. The EDS spectra of the samples as well as the Zn to La ratios are presented in Figure 4.

The initial precipitation mixture of salts contained Zn:La in a 1:0.25 ratio, while in the surface of the as-synthesized form, the ratio of Zn:La reached only 1:0.138. It shows that the La ions precipitation extent was not as high as the Zn incorporation in the product. The Zn: La ratio further decreased by the calcination procedure, showing that some of the La from the surface diffused inside the solid particles. Since the lanthanum-containing phases are only barely seen in the XRD patterns and however the La proportions highlighted by the EDS were a lot higher than those from the work of Maninkandan [17], it results in a big number of nanoparticles of lanthanum oxide and-or lanthanum hydroxide on the surface, too small to give signals in the XRD patterns. The morphology and composition changes associated with the calcination generate phase boundaries [26] very favorable for conferring superior capacities of electron-hole pair stable generations and consequently higher photocatalytic activity (see Section 3.4).

### 3.2. BET Analysis

**Figure 5:**
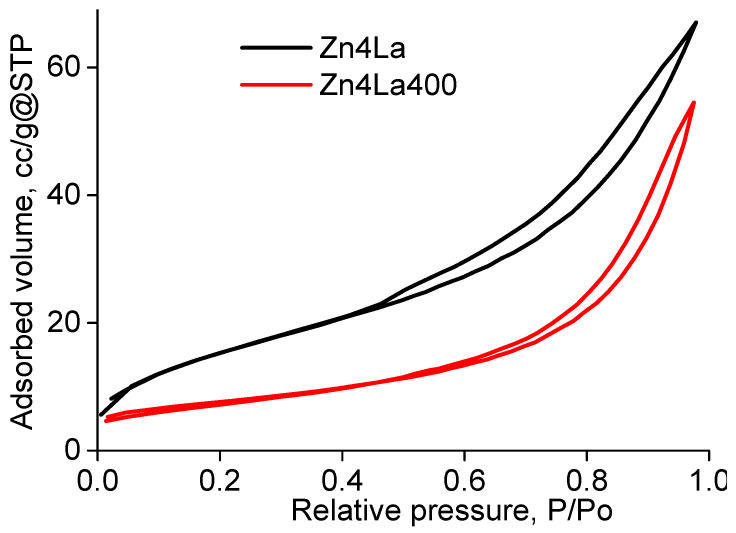
BET isotherms of the samples.

The porous structure of the solids was investigated by the adsorption of pure nitrogen at −196 °C. The values of the specific surface obtained by applying the BET model (proper for multilayer physic-sorption) were, respectively, of 56.04 m^2^/g (Zn4La) and 26.34 m^2^/g (Zn4La400) and the corresponding free pore volumes, determined at the highest value of the P/P_o_ value are, respectively, of 0.104 cm^3^/g (Zn4La) and 0.084 cm^3^/g (Zn4La400). These data are in line with the results reported in literature for oxides similarly prepared [27,28,29,30]. The calcination procedure generally determines the decrease of both specific surface area and free pore volume, due to the structure compacting associated with the advanced crystallization process or to the collapse of the “soft” architecture of the solids obtained by precipitation at mild temperatures. The adsorption isotherms are displayed in Figure 5.

The shapes of the adsorption isotherms resemble to a certain extent to the isotherm type II, according to the IUPAC classification [31,32] indicating a solid with porous structure, able to adsorb in multilayers, having a quite uniform surface. More recently, new definitions about “typical” porosity features were completed by the information brought by the examination of the hysteresis loop from the desorption branch. The loops allures were also classified in six types [33]. Our samples display mostly a H3 loop type: the desorption branch is almost parallel with the adsorption branch for most of the range between 0.95 and 0.45, then the loop shrinks and the branches of the isotherm unite each other. This behavior is characteristic for the non-rigid aggregates of plate-like particles which define macropore networks incompletely filled with liquid nitrogen.

**Figure 6:**
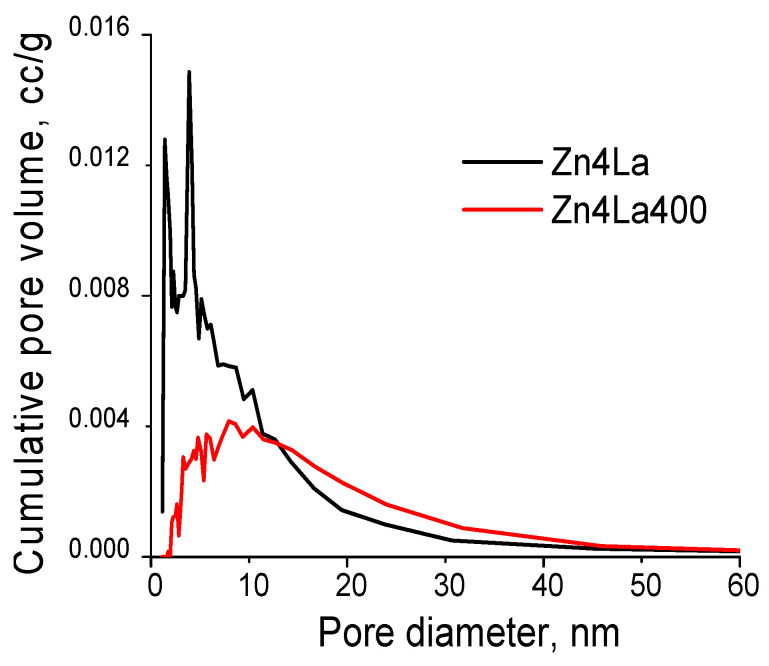
Pore size distribution of the samples obtained by applying the BJH model.

The pore size distribution, calculated by using the BJH model [34] revealed a very wide and patchy range of pore sizes, which is typical for solids formed by the abundant association of platelets (Figure 3). In these conditions, qualitative evaluation shows that the pores become wider after the calcination but a characteristic pore size cannot be defined (Figure 6). 

### 3.3. FTIR Analysis

**Figure 7:**
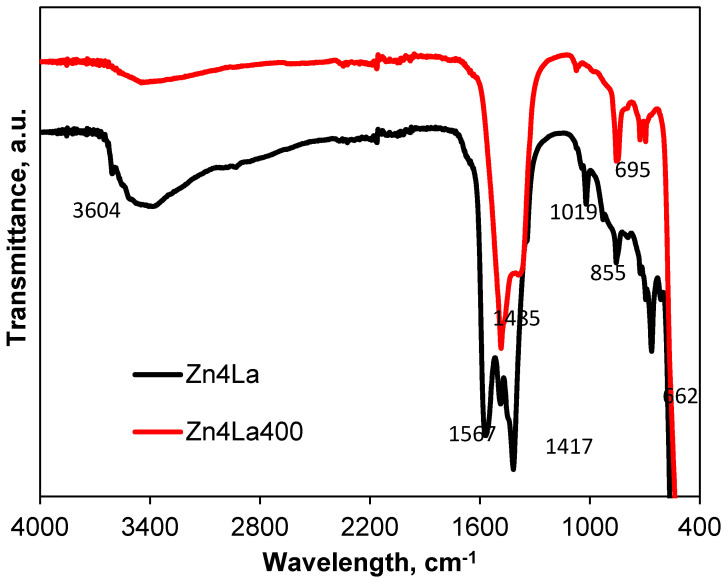
Infra-red (IR) spectra of the samples.

The Fourier-transform infrared (FT-IR) spectra of the obtained samples are shown in Figure 7. The spectrum of the initial sample Zn4La shows a sharp band from 3604 cm^−1^ attributed to the OH groups from the La(OH)_3_. The wide band centered at about 3450 cm^−1^ is assigned to the water molecules incorporated in the solid (ν_H2O_) and coexists with the deformation band (δ_H2O_) from 1567 cm^−1^ which disappears by heating/annealing at 400 °C. The bands located at 1485 cm^−1^, 855,662 cm^−1^ correspond to the formation of La_2_O_3_ mixed with LaO (OH) [35,36]. This is supported also by the disappearance of the band from 1019 cm^−1^, the modification of the bands from 1417 and 695 cm^−1^, which become less intense and suffer modifications of the aspect. The presence of ZnO is confirmed by the broad band located in the range 500–400 cm^−1^ [37].

### 3.4. Band Gap

A relevant characteristic of a semiconductive oxide endeavored to be used as photocatalyst is the value of the band gap energy necessary to promote the electron jump from the valence band to the conduction band. This process is the key for the formation of an electron-hole pair in the structure of a semiconductive material, each of the two ionic entities being able to initiate reactions with water and oxygen and form hydroxyl radicals HO^•^, responsible for initiating the oxidation reactions of persistent organic pollutants (POPs). 

The measurement of the band gap value (*BG*) is possible using the diffuse reflectance spectra, by extrapolating of the linear portion of the spectrum up to cross the x axis and convert the corresponding wavelength (nm) in energy, by using the relation:

*BG* (eV) = 1240/*λ* (nm). (3)

**Figure 8:**
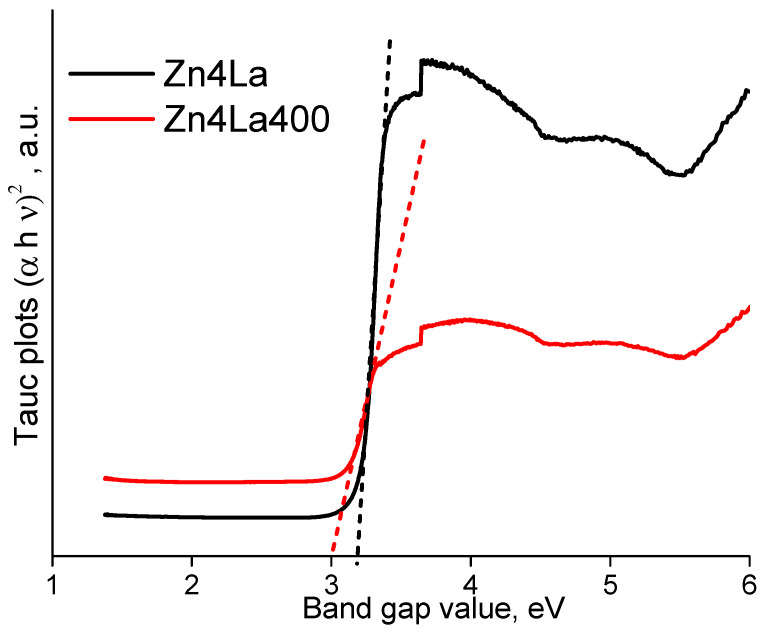
Tauc plots for the Zn4La and Zn4La 400 samples.

For improving the accuracy of *BG* value measuring, mathematical processing is applied by defining the Tauc function, showing that there is a proportionality between (*αhν*)^1/n^ ∝(*hν*—*BG*), where α is the adsorption coefficient of the material, h the Planck’s constant, ν the frequency of activating light and Eg is the band gap energy. The value of n depends on the electronic nature of the band gap, having values corresponding to the possible transitions types as follows: 3 for indirect forbidden (IF), 2 for indirect allowed (IA), 3/2 for direct forbidden (DF) and 1/2 for direct allowed (DA) transitions, respectively [38,39,40]. The graphical representation of (αhν)^2^ versus hν (the electron energy) for direct transition of electrons allows to determine the BG value, by extrapolating the Tauc function till crossing the x-axis (Figure 8).

The band gap value changes from 3.23 eV in the case of the uncalcined sample Zn4La to 3 eV for sample Zn4La400.

### 3.5. Photocatalytic Activity Investigations 

#### 3.5.1. Preliminary Experiments: Role of Adsorption and Photolysis

**Figure 9:**
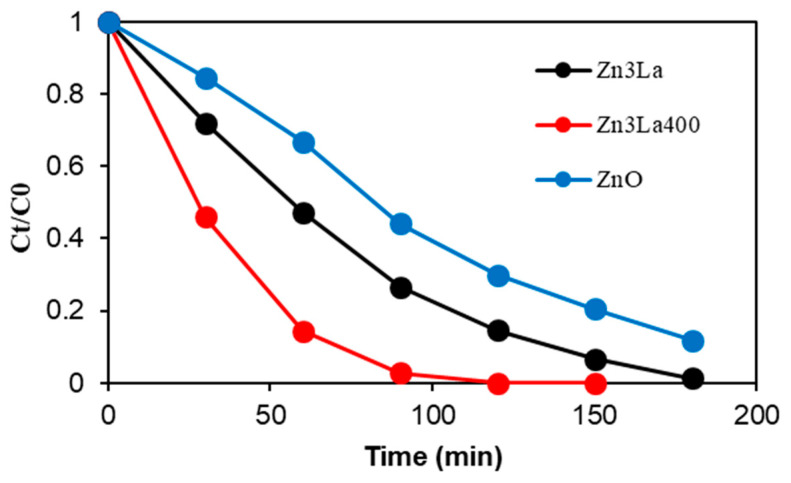
Photocatalytic activity of the prepared samples in clofibric acid (CA) degradation compared to pure ZnO (catalyst load 500 mg/L, CA concentration 20 mg/L, natural solution pH, stirring rate 450 rpm and UV-A irradiation).

Preliminary photocatalytic studies were carried out to compare the photocatalytic activity of the synthesized nanostructures with pure ZnO. The experiments were conducted using 500 mg/L, at ambient temperature and under UV-A irradiation conditions. As stated in the introduction section, clofibric acid, an organic compound frequently found in the effluents of wastewater treatment plants, showing high persistence in the environment, was employed as model molecule. Aqueous solutions having an initial pollutant concentration of 20 mg/L were used. For comparison purposes, the results obtained for both samples are depicted in Figure 9.

It is observed that the photocatalytic activity of these materials is quite different. Accordingly, the almost total (97%) pollutant elimination was achieved in the presence of Zn4La400 after an irradiation time of 90 min, while only 73% were degraded with Zn4La, proving the considerably higher photocatalytic activity of the calcined material. In addition, it was found that the synthesized materials exhibit a higher reactivity compared to pure ZnO confirming the positive role of La doping on the photocatalytic activity of ZnO materials, as well as the efficiency of the coprecipitation method employed for their synthesis. A similar effect was previously found by Nguyen et al. and Sakir et al. for lanthanum doped nanoparticles in the degradation of methyl orange and paracetamol, respectively [13,16].

It should be noticed that the comparative investigation of both materials conducted in dark to check the adsorption effect on the elimination of CA showed only a very low variation in the pollutant concentration (data not shown). It is very important to underline that from the point of view of the specific surface area values, in connection with the adsorption of CA and its transformation on the surface, we could expect that the Zn4La behaves better than Zn4La400 since its specific surface is more than double, which was in contradiction with the practical obtained results. It is thus very important to observe that even the lower specific surface of the Zn4La400 sample is attactive enough to ensure a saturation with adsorbed CA and that the superior ability of this solid to form oxidant species (HO^•^ radicals) by the irradiation was the success key in the photocatalytic process. Indeed, the easier and more effective formation of the electron-hole pair reflected by the lower band gap value of Zn4La400 sample was also retrieved in its better behavior in the reaction.

The results depicted in Figure 9 are only related to the photocatalytic performance (after reaching the adsorption-desorption equilibrium). Moreover, our findings also confirm that the calcination step plays a favorable role on the photocatalytic reactivity of these structures. Different studies reported the beneficial role of calcination on the physico-chemical properties of synthesized materials and on their photocatalytic activity [41,42,43]. Basically, the main action of the thermal process is to induce the elimination of some volatile compounds (water, carbon dioxide or organic molecules) from the prepared sample but in most cases, this treatment is employed to induce a changement of the physico-chemical properties of a material or to its composition (for example transformation of metal to its metal oxide) [44].

According to these results, the calcined sample Zn4La400 is more photoactive with respect to Zn4La, therefore it will be considered for further photocatalytic investigations.

#### 3.5.2. Effects of Some Operating Conditions on the Clofibric Acid Elimination with Zn4La400

**Figure 10:**
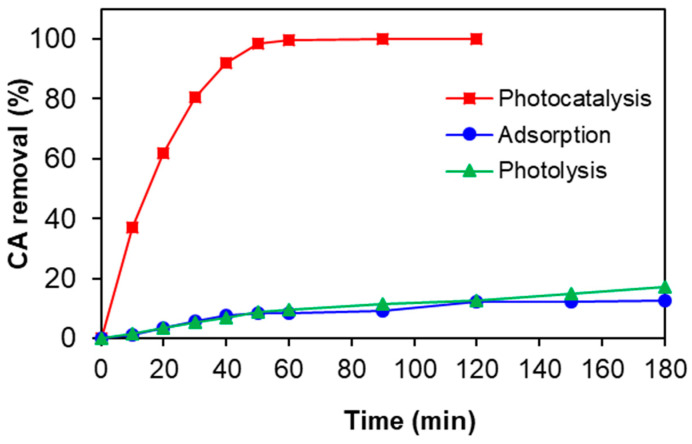
Comparison of the CA elimination using Zn4La400 during adsorption, photolysis and photocatalysis (500 mg/L Zn4La400, initial pollutant 3 mg/L, stirring rate of 450 rpm and UV-A irradiation).

Few blank tests were firstly carried out in batch system, under maximal irradiation conditions and without catalyst, to determine if the direct photolysis affects the elimination of this pollutant. For example, the typical results corresponding to the experiments performed at 3 mg/L of pollutant, 500 mg/L of catalyst and for natural solution pH are depicted in Figure 10. It was found that the CA removal in the absence of catalyst is of only less than 9% after 60 min and 17% after an exposure time of 180 min. Similar results were previously reported in other papers [45,46,47].

On the other hand, preliminary adsorption experiments were also conducted in dark under different conditions (mass of catalyst and pollutant) to assess the elimination of CA during this process and to determine the contact time necessary to achieve the adsorption-desorption equilibrium. According to the results, a very low sorption affinity of Zn4La400 towards the target molecule was found for all the investigated conditions. For comparison purpose, only the results corresponding to the test conducted at 3 mg/L of pollutant and catalyst content of 500 mg/L are presented in Figure 10. A quite similar behavior in terms of CA elimination efficiency is noticed in this case in comparison with direct photolysis. Thus, the adsorption is not a determining mechanism in the removal of this molecule. Other authors reported about the poor adsorption capacity of this molecule in the presence of titanium dioxide as photocatalyst [45,47,48]. Moreover, it can be noticed that 60 min of contact time in dark was enough to complete the adsorption equilibrium under all the investigation conditions. The results from the same figure also reveal that the removal of pollutant is strongly enhanced in the presence of the Zn4La400 and UV-A irradiation, confirming the efficiency of this solid and the positive role of photocatalysis in the elimination of target compound. The initial CA was completely photodegraded over the considered catalyst load after a reaction time of 60 min, which is very promising, considering the recalcitrance of this molecule. Taking into account the presented data, it is obvious that the elimination of clofibric acid is exclusively caused by the interaction between Zn4La400 and UV-A irradiation, while the role of other possible mechanisms such as direct photolysis or adsorption in the removal of this molecule can be considered as insignificant.

**Figure 11:**
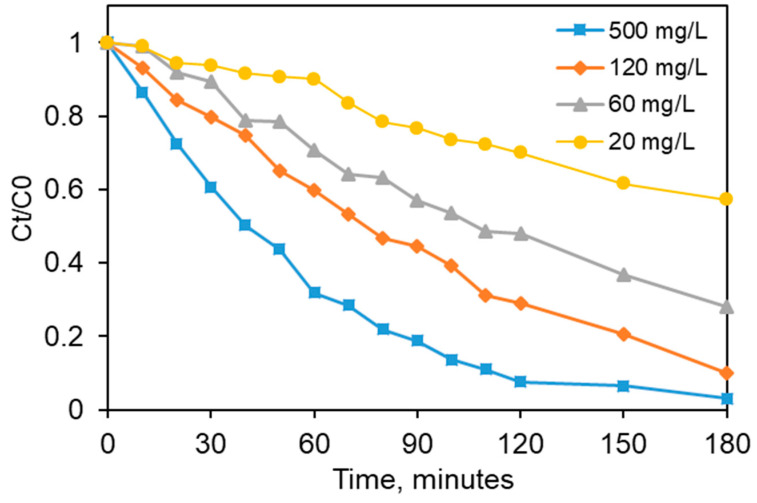
Effects of the catalyst load on the photodegradation of clofibric acid (pollutant concentration 10 mg/L, stirring rate 450 rpm, natural solution pH, UV-A irradiation).

In a photocatalytic process, the catalyst loading is a key aspect to be considered, because of its effect on the pollutant oxidation rate. In addition, this parameter dictates the economic viability for a possible future process implementation [49,50]. For these considerations, it is of utmost importance to obtain more insights about the effect of this parameter on the photocatalytic degradation efficiency. In this context, some experiments were carried out in aqueous suspension by varying the photocatalyst dose from 20 mg/L to 500 mg/L. The other process factors such as initial pollutant concentration (10 mg/L), irradiation conditions and solution pH were kept constant. Figure 11 shows the obtained results for the effect of the catalyst concentration on CA residual content against the reaction time. It was found that the pollutant elimination strongly increases with the photocatalyst load, showing a maximal pollutant degradation rate for a catalyst content of 500 mg/L. For a low catalyst concentration (i.e., 20 mg/L), only 43% of pollutant elimination was achived after 180 min of irradiation. A similar trend was noticed in previous literature studies in the case of other catalysts [51,52,53].

Such a behavior is specific for the photocatalytic systems, because the increase of catalyst content increases the number active sites available at the catalyst surface which determines the photons absorption and thus the photodegradation efficiency [54]. Another possible explanation is that, for may organic compounds, their photocatalytic decomposition follow the surface-adsorbed reactants isotherm (Langmuir model). In this case, the degradation of these molecules is basically proportional to the number of reactant molecules adsorbed on the catalyst surface [55]. The exceeding of an optimal limit of the photocatalyst concentration could bring theoretically speaking more available active sites but the yield of irradiation capturing decreases, due to the fact that the turbidity of the suspension increases so much that the irradiation cannot reach all the available sites from the surface of the semiconductive photocatalyst [56].

The results obtained in our study indicate that for the concentrations range employed in the experiments, the best values in the case of Zn4La400 were obtained at a dose of 500 mg/L. Since the differences in CA degradation yields at photocatalyst doses between 120 and 500 mg/L are not very large, we could not expect spectacular improvements at higher doses. Moreover, increasing the dose brings a decay of the economical value of the process envisaged, due to the cost of the photocatalyst. Also, the literature mentions situations when an excedent of solid in suspension during the photocatalytic reaction decreases the degree of radiation absorption due to the too level of turbidity in the system. Therefore, the value of 500 mg/L was chosen to be used for the next photocatalytic tests.

**Figure 12:**
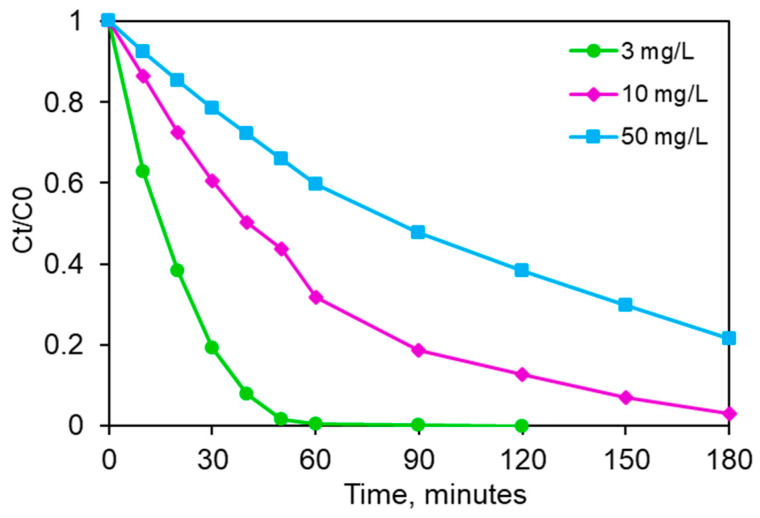
Effect of the initial clofibric concentration on the photocatalytic degradation efficiency (Zn4La400 500 mg/L; stirring rate 450 rpm, natural pH, UV-A irradiation).

Another parameter which can affect the photocatalytic activity of the considered catalyst is the initial pollutant concentration. Thus, the oxidation of the target molecule over the Zn4La400/UV-A system as a function of the initial clofibric acid concentration was also considered in this work. Figure 12 presents the obtained profiles for the CA elimination as a function of its initial CA content in the solution. As expected, a certain decrease in the pollutant elimination was found with the incease of CA concentration. For the lowest investigated concentration, 3 mg/L, only 60 min of irradiation were necessary to remove completely CA from the reaction media. In the meantime, the degradation rate were, respectively, around 70% for 10 mg/L CA and 40% for 50 mg/L, respectively after 60 min. These results are consistent with the one previouly reported by Vrinceanu et al. [45], Favier et al. [48] and Burlacu et al. [57] for the same molecule using two commercial catalysts, named P25 Aeroxide^®^ and ZnO and UV-A irradiation conditions. However it should be noticed that a real comparison of these data in terms of photodegradation efficiency is difficult because of the existing difference between the operating conditions (catalyst loading, reaction time, intitial pollutant concentration, etc.), reactor geometry and irradiation source. On the other hand, it should be highlighted that in terms of clofibric acid photocatalytic degradation to the best of our knowledge the literature data is relatively scarce.

The behviour observed in our study may be attributed to the fact that for a given catalyst loading and incident light irradiation, a nearly constant concentration of photogenerated active species will be generated in the solution and, in function of the initial pollutant content in the reaction mixture, they will or not be a limiting factor for the photocatalytic process [54]. The data obtained in this work suggest such a reactant limitation on the pollutant on the degradation overall. It should be kept in mind that some of the potential adsorption sites for CA from the photocatalyst remain for a time unavailable for the adsorption, since the primary degradation products are formed during the reaction on the surface, in adsorbed state and a delay appears in rendering the active sites free from them by desorption. This effect is more pronounced at high initial concentrations [50].

**Figure 13:**
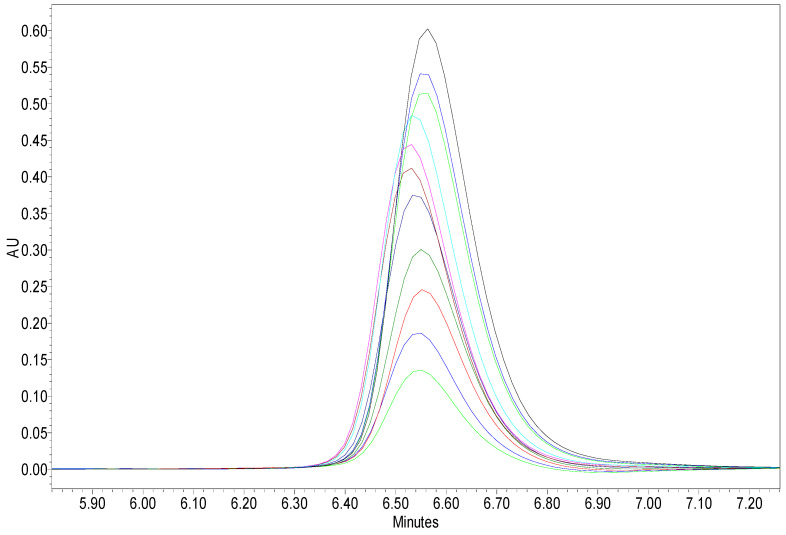
High performance liquid chromatography (HPLC) chromatograms collected during a photocatalytic experiment showing degradation efficiency of CA in the presence of Zn3La400 under UV-A irradiation as well as the formation of reaction intermediates and = 500 mg/L; [CA] = 50 mg/L.

The results collected in our study demonstrated the photocatalytic activity of the considered catalyst and the target compound degradation. The pollutant concentration was measured by high performance liquid chromatography (HPLC) and was detected at 227 nm. The pollutant elimination during the photocatalytic reaction was really confirmed by the decrease of the chromatographic peaks of the samples collected during the photocatalytic process. For illustration purposes, Figure 13 displays the HPLC results for the experiment conducted at 50 mg/L CA accordingly, a dramatic decrease of the measured absorbance with irradiation time was observed demonstrating the rapid degradation of this molecule by Zn4La400/UV-A system. 

It should be noticed that photocatalysis is today recognized as one of the most effective strategies for water purification. The main interest of this process consists in its mineralization ability because of the reactive species generated during the photocatalytic reaction. Because of their high oxidation potential, these species are able to transform and mineralize different organic molecules into CO_2_ and inorganic ions. For this reason, in order to check the photocatalytic efficiency of Zn4La400 catalyst a special attention was paid to the evaluation of its mineralization performance. 

Table 2 gives the results found for the TOC removal yields (mineralization) of CA for different initial pollutant concentrations and 500 mg/L of catalyst. For the low CA concentration of 3 mg/L, a removal of about 71% was achieved after 180 min of reaction. A further increase of the contact time could probably enhance the pollutant mineralization.

The mineralization efficiency decreases for 44% to 30% with the increase of initial pollutant concentration from 10 to 50 mg/L, respectively. However, these values are much lower than the degradation yields determined by HPLC, because of the high content of reaction intermediates render more difficult to complete the mineralization. In some works, such a behavior is explained by the fact that, for a low pollutant amount, the active sites which are present at the catalyst surface are in excess for both accommodating the pollutant molecules and remain free for radiation capture and radicals generation. On the contrary, when the initial pollutant concentration increases, less reactive radicals are generated, because under these conditions the catalyst surface remains occupied by more molecules of pollutant and reaction byproducts and, thus, fewer sites remain available for the generation of the highly active oxidants [45,58].

The results obtained in our study are very promising in terms of pollutant degradation and mineralization, providing that designed mixed oxides can be considered as an interesting catalyst for future potential photocatalytic applications.

### 3.6. Investigation of the Catalyst Recyclability

**Figure 14:**
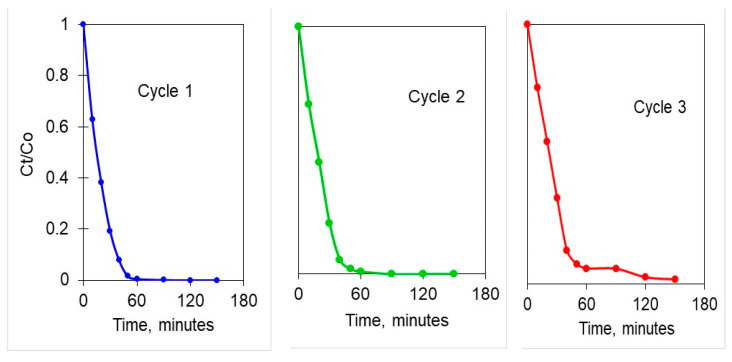
Clofibric acid elimination with Zn4La400 during three consecutive cycles (catalyst load 500 mg/L, initial pollutant concentration 3 mg/L, natural solution pH, stirring rate of 450 rpm, UV-A irradiation).

In order to check the potential use of a catalyst in a photocatalytic process, other fundamental aspects such as the catalyst longevity in the reaction and its recovery are necessary to be considered in addition of its photocatalytic activity [59,60,61]. Indeed, the recyclability of catalyst is necessary to be studied in order to check its reusability capacity and estimate the possible application of a semiconductor in a heterogeneous photocatalytic technology [62]. In the light of these facts, in our study we focused our attention on the evaluation of this aspect. Three consecutive photocatalytic experiments employing fresh solutions of clofibric acid were conducted to evaluate the photocatalytic stability of Zn4La400 for the degradation of this molecule. They were carried out for 500 mg/L of catalyst, 3 mg/L of pollutant and at a natural solution pH. After each cycle, the catalyst was separated by filtration, rinced two times with ultrapure water, dried overnight at 110°C and then used in a new photocatalytic experiment. Each cycle was conducted using the same experimental protocole. According to the obtained results (Figure 14), for the first run conducted in the presence of the fresh catalyst the pollutant was completely removed after 60 min of reaction.

In the second cycle, the photocatalytic activity of Zn4La400 remains unchanged and only a slightly loss of its activity (6%) was noticed after the third photocatalytic cycle after 60 min of reaction. These results are in line with the ones reported previously [45] for the same molecule using P25 Aeroxide^®^ titania. Some researchers claimed similar results for the photocatalyst stability in the degradation of different organic molecules using other catalytic supports [54,58].

The observed slight decrease in the photocatalytic activity of Zn4La400 can be attributed to a loss of material during the catalyst separation or washing steps, as well as to the blokage of some of the active sites by the remaining fragments resulted during the degradation during the oxidation reactions. In conclusion, these findings reveals that the used catalyst preserved almost totally its photocatytic activity in the degradation of a highly stability molecule, which makes it quite promising in real applications.

## 4. Conclusions

A mixed oxide material containing zinc and lanthanum in oxide form was prepared by the coprecipitation from a solution containing salts of both metals dissolved in ultrapure water, at a constant pH of 10, by using NaOH solution (1 M). The resulted solid was characterized by XRD, SEM/EDAX, IR, UV-DR and BET adsorption of pure nitrogen. The as-synthesized product consisted of a mixture of zinc oxide, lanthanum hydroxide and lanthanum oxide. Upon calcination at 400 °C, the lanthanum hydroxide was transformed in lanthanum oxide and the outer layer became poorer in lanthanum, due to its diffusion inside the particles. This transformation brought the diminution of the specific surface two times and a slight decrease of the band gap value.

The photocatalytic properties of the fore-mentioned samples were tested in the removal of clofibric acid (CA), from diluted solutions to evaluate their activity. Despite the decrease of the specific surface area value, the calcined sample displayed much better photocatalytic performance, revealing that the formation of the oxidant species promoted by the lower band gap value exerts a much consistent influence on the overall process than the opportunities of adsorption connected to high specific area values. The formation of the reactive oxidizing species from promoted electrons and/or holes and water proves to be the determining reaction step, the availability of adsorbed molecules being easier-to-reach.

Detailed investigations concerning the photocatalytic performance were performed on the calcined sample. The most advantageous photocatalyst dose was found to be or 500 mg/L. The decomposition yield strongly depends on the CA concentration. At a 3 mg/L CA, the decomposition was complete in 60 min and furthermore, the activity was almost totally preserved after three cycles of use of the same solid. The results obtained at more concentrated CA solutions also revealed that the solid is very active: more than 93% yield was obtained after 150 min starting from 10 mg/L, while almost 80% was degraded in 180 min from a solution of 50 mg/L.

The mineralization degree measured by TOC fallen between 72% from the CA solution of 3 mg/L and of 29.5% from 50 mg/L, after 180 min of photocatalysis.

These results recommend this photocatalyst as suitable especially for the advanced removal of trace concentrations of persistent organic compounds from water.

## Figures and Tables

**Table 1:** Comparison between Zn and La atoms and ions size.

Species	Atomic Radius, Å	Ionic Radius, Å
Zn	1.53	0.74
La	2.74	1.16

**Table 2:** TOC removal in the presence of Zn4La400 (500 mg/L) catalyst after 180 min of UV-A irradiation.

Pollutant Concentration, (mg/L)	TOC Removal, (%)
50	29.5
10	44.2
3	72.1
